# Editorial: Impact of the Coronavirus Pandemic (COVID-19) on Mood Disorders and Suicide

**DOI:** 10.3389/fpsyt.2022.846112

**Published:** 2022-01-28

**Authors:** Chiara Ciacchella, Virginia Campedelli, Giorgio Veneziani, Gaia Romana Pellicano, Daniela Sambucini, Carlo Lai

**Affiliations:** Department of Dynamic and Clinical Psychology and Health Studies, Sapienza University, Rome, Italy

**Keywords:** COVID-19, pandemic, mood disorders, suicide, mental health

The unpredicted spread of the Coronavirus (COVID-19) led to a global crisis that radically changed our lives. After nearly 2 years of the pandemic, the COVID-19 still represents a significant threat to individual and global safety. The governments have adopted necessary restrictive measures to contain the infection and reduce the impact of the crisis on healthcare systems worldwide, forcing people to socially distance and isolate. For this reason, the global outbreak still has important repercussion not only on the physical health: psychological well-being has been severely impacted considering that during the pandemic period the risk of the mental disorder onset also increased.

The 40 papers brought together in this Research Topic provide information about the overall effects of the COVID-19 outbreak on mood disorders and their behavioral consequences. The studies focused on the main risk factors associated with the development of depression, anxiety, and suicidal ideation in different countries and populations (e.g., healthcare workers, students, and people with specific clinical conditions). Moreover, the Research Topic highlights current challenges to cope with the psychological impact of COVID-19, providing insight for the clinical practice to support healthcare professionals, patients with COVID-19, and their relatives.

One of the main consequences of the COVID-19 was the growing prevalence of the depression and anxiety symptoms, which seemed to be higher than the one reported during previous pandemic events, as well as the outbreak of SARS (1, 2), MERS (3), and Ebola (4) (Gong et al.), across the globe. The study of Han et al., conducted in China, showed greater severity of depression when compared with a pre-pandemic period. These findings are consistent with the data related to Australia (Dawel et al.), Wales (Gray et al.), Italy (Bussone et al.), Libya (Elhadi et al.), United States (Rodriguez-Seijas et al.), and Arabia (Khoshaim et al.). Moreover, the impact of COVID-19 influenced sleep quality, as reported by several studies that found an alarmingly high prevalence of insomnia [(5), Bacaro et al.].

A relevant issue was the risk of suicide, as reported by a recent meta-analysis which reported increased event rates for suicidal ideation, suicide attempts, and self-harm during the COVID-19 pandemic (6). It is relevant to consider that the pandemic was not related exclusively to a greater risk of the overmentioned symptoms, but it had an important role in the worsening of the psychological state of people with pre-existing psychiatric conditions and other medical diseases (e.g., bipolar disorders, tourette syndrome, multiple sclerosis, and postpartum depression) (Carmassi et al.; Conte et al.; Donisi et al.; Gobbi et al.; Spinola et al.). In fact, the management of other diseases was affected by the reduction of health system resources which were used to cope with the pandemic crisis.

Considering the prevalence of the presented clinical conditions, several risk factors have been investigated. The forced isolation, caused by the strict measures adopted to contain the spread of coronavirus, had transformed day-to-day life, reduced social interactions, and increased fear and anxiety about COVID-19. The adverse effects of the sudden lockdown were particularly evident in the younger generation (Khoshaim et al.; Saravanan et al.). Indeed, the prevalence of symptoms of anxiety and sleep problems was high in university students who experienced uncertainty about their future due to the unpredictable course of the pandemic (Wang et al.). The lockdown was associated with increased feelings of loneliness and poor perceived social support, which seemed to play an important role in the development of depressive and anxious symptoms, increasing the risk of suicidal ideation (Boursier et al.; Cheung et al.; Hoffart et al.; Raj et al.; Velotti et al.). Rumination and COVID-19 related fear could lead to negative affect (Bachem et al.) and could increase feelings of loneliness (Hoffart et al.). Potentially, the relationship between solitude and poor mental health might be mediated by emotion regulation strategies (Velotti et al.), considering that the distance from significant relationships could result in emotional regulation difficulties (Mariani et al.). In this regard, family support plays an important role in reducing the negative impact of lockdown and loneliness, acting as a protective factor against stress (Mariani et al.). Social interactions were carried out with social media and virtual communities to cope with loneliness and negative emotions, providing a slight relief from distress (7). However, the frequency of mobile phone dependence in college students and adolescents was higher than before [(8); Muzi et al.]. It must be pointed out that the excessive use of social media, prolonged in time and forced by the pandemic situation, was associated with increased levels of anxiety (Boursier et al.). Otherwise, staying physically active would attenuate the side effects of COVID-19 on mental health, reducing the risk of the onset of depression and anxiety (Hu et al.).

Another important factor that affected mental health during the outbreak was the employment status, as observed by Mojtahedi et al. Indeed, people who lost their jobs during the pandemic showed higher levels of negative affective states (Mojtahedi et al.).

The Research Topic highlighted the significant impact of the COVID-19 on the healthcare workers (HCW). The HCW faced intense workloads, more significant distress, and a higher risk of occupational exposure (Jaiswal et al.; Li et al.). This professional category experienced high levels of psychological distress due to the overwhelming working environment and the frequent risk of contact with the virus [(9), He et al.; Sirois and Owens]. Moreover, the worry about transmitting the infection to their relatives (Fageera et al.; Sirois and Owens) contributed to increase the tension and to strengthen the feelings of loss of control. In this context, the nurses in intensive care units (ICU) seemed to be the category affected the most (Li et al.), showing high rates of depression and anxiety (Fageera et al.).

Noteworthy, some “psychological antibodies” have been identified as valuable elements to safeguard the mental health of HWC, such as life satisfaction and well-being dimensions (personal growth, self-acceptance, and positive relations) (Jaiswal et al.). Furthermore, long professional experience, adequate training, and clear guidelines, information, and protocols for infection control have been found to buffer the psychological impact of the pandemic (Fageera et al.; Sirois and Owens). Additionally, adequate formal or informal support from supervisors and co-workers was important for reducing stress among HCW (Sirois and Owens).

In light of the results gathered from studies on this Research Topic, there is an evident need to plan interventions to prevent anxious and depressive symptomatology and mitigate the negative psychosocial consequences of COVID-19 (Ge et al.) by improving emotional regulation skills and reducing isolation from family (Mariani et al.). In this regard, Kong et al. showed the effectiveness of a psychological-behavioral intervention in patients with COVID-19. Frequent communication with medical staff and the psychological support provided to hospitalized patients seemed to alleviate the anxiety and fear caused by the virus (Kong et al.). Moreover, the appraisals related to COVID-19 may play a vital role in the psychological well-being of infected patients. Therefore, supporting the readjustment to society among COVID-19 patients is relevant and must be considered when promoting mental health recovery (Chen et al.).

Finally, Bachem et al. underlined the pivotal role of the institutions in an individual's mental well-being during a situation as a pandemic, where being supported by the authorities is an important source of psychological relief. Therefore, special attention should be paid to strengthening trust in the institutions, as this can alleviate the negative impacts of COVID-19 related fears (Bachem et al.).

In conclusion, the pandemic crisis is still ongoing, and uncertainty about the future could worsen people's mental health. New restrictive measures could be periodically imposed, fostering further feelings of loneliness, and exacerbating psychological disorders. In addition, new challenges are opening up related to the emotional burden management, socio-economic crisis, and vaccination. Thus, it is important to plan psychological intervention strategies that can help the general population to cope with the emergency, as well as to address the specific needs of young adults, health care workers, and patients with medical diseases.

## Author Contributions

CL, CC, VC, and GV wrote the editorial. CL, GP, and DS conceived the editorial and supervised the work. All authors read, performed critical revision, and approved the final version of the editorial.

## Conflict of Interest

The authors declare that the research was conducted in the absence of any commercial or financial relationships that could be construed as a potential conflict of interest.

## Publisher's Note

All claims expressed in this article are solely those of the authors and do not necessarily represent those of their affiliated organizations, or those of the publisher, the editors and the reviewers. Any product that may be evaluated in this article, or claim that may be made by its manufacturer, is not guaranteed or endorsed by the publisher.
